# Epigenetic basis of cancer drug resistance

**DOI:** 10.20517/cdr.2020.06

**Published:** 2020-03-19

**Authors:** Moammir H. Aziz, Aamir Ahmad

**Affiliations:** ^1^James H. Quillen VA Medical Center, Johnson City, TN 37604, USA.; ^2^School of Medicine, University of Alabama at Birmingham, Birmingham, AL 35205, USA.

Cancer is a deadly disease and resistance to therapies is a major reason that renders it particularly lethal. A majority of research thus far has focused on genetic factors that form the basis of cancer drug resistance. However, it is increasingly being realized that epigenetic regulation plays a very important role in determining the resistance of individual tumors to certain therapies. Several novel drugs that target epigenetic events are under investigation, thus serving as a testimony to the enormous potential of exploiting epigenetics in tackling the problem of cancer drug resistance. Moving forward, a focus on epigenetics will be critical for a better understanding of the phenomenon of cancer drug resistance and for the design of novel therapies.

Some cancer patients are inherently resistant to specific therapy because of their genetic makeup (*de novo* cancer drug resistance) while other cancer patients initially respond to therapy, but eventually develop resistance with continued administration (acquired cancer drug resistance)^[1]^. A good understanding of cancer drug resistance is critical to the efficient management of cancer patients in the clinics. Epigenetic events have a profound effect on the onset as well as progression of human cancers^[2,3]^. Methylation and acetylation are two well-studied epigenetic events that are known to profoundly affect the expression of genes, resulting in activation of oncogenes and/or suppression of tumor suppressor genes, leading to development of cancer drug resistance. DNA methylation, histone modifications (methylation, acetylation, phosphorylation, ubiquitylation, sumoylation, *etc*.), and regulation through microRNAs (miRNAs) are some of the active areas of cancer research, encompassing the epigenetic regulation (see the Special Issue on: Non-coding RNAs in Response and Resistance to Therapy in Cancer).

The tyrosine kinase inhibitors (TKIs) have an important role in cancer therapy. They are effective against many cancers, both solid and liquid. They have particularly been used to inhibit the oncogenic activity associated with epidermal growth factor receptor (EGFR), a receptor tyrosine kinase. However, acquired resistance against EGFR-TKIs is well-studied^[4]^ with reports on its epigenetic regulation^[5,6]^. White *et al*.^[7]^ reviewed the subject by evaluating a role of histone lysine demethylases in resistance against TKIs. Up-regulation of these demethylases leads to resistance against TKIs. For example, amplification of *KDM5A* gene in breast cancer correlates with erlotinib resistance, as well as with gefitinib resistance in lung cancer. Erlotinib and gefitinib are first generation EGFR-TKIs. Similarly, KDM1A, also known as lysine-specific histone demethylase 1A, mediates resistance against sorafenib in liver cancer cells. Sorafenib is a kinase inhibitor effective against rapidly accelerated fibrosarcoma, vascular endothelial growth factor receptor, and platelet-derived growth factor receptor kinases. Based on this evidence, inhibition of lysine demethylases can be an effective strategy to overcome resistance against TKIs.

Ovarian cancer is the most lethal gynecological cancer, with a reported epigenetic basis of drug resistance^[8]^. Platinum-based anticancer drugs are commonly used to treat ovarian cancer but the tumors often progress within the first two years of treatment. Schwarzenbach and Gahan^[9]^ discussed the epigenetic signatures, such as DNA methylation, histone modifications, and miRNA deregulation, in cis- or carboplatin resistant ovarian cancers. Such knowledge can help identify patients who will respond favorably to platinum-based therapies *vs*. those who will not. This is a step towards personalized medicine in cancer, a concept that is increasingly attracting the attention of cancer researchers^[10-12]^. Breast cancer is another cancer that is frequently diagnosed in females and which remains a leading cause of cancer-related deaths in females worldwide. Among the many mechanisms, epithelial-to-mesenchymal transition (EMT) and the acquisition of cancer stem-like phenotype provide plasticity to breast cancer cells, rendering these cells highly invasive, metastatic, and resistant to therapies. The article by Ponnusamy *et al*.^[13]^ provides a better understanding of epigenetic modifications underpinning the cellular reprogramming, particularly the EMT and stem cell phenotype. Such knowledge is essential to design future therapies to overcome cancer drug resistance. On a similar topic, the research article by Seno *et al*.^[14]^ describes the cytotoxic action of daunorubicin, a topoisomerase II inhibitor, against mouse induced pluripotent stem cells-derived cancer stem cells.

The relevance of epigenetic events is not limited to solid tumors. In acute lymphoblastic leukemia, a common childhood malignancy, there is an increasing realization that genetic changes are not enough to describe the observed chemoresistance and the resulting relapse. As a result, researchers have started looking at the epigenetic changes as a possible answer to this problem^[15]^ and this is the topic of discussion in the article by Meyer *et al*.^[16]^. Further, a cell organelle that is relatively less studied in the context of epigenetic regulation of drug resistance is the centrosome. The centrosome plays a very important role during cell division, ensuring proper distribution of cellular components among daughter cells. Any aberrant functioning of centrosomes, through epigenetic events, can compromise the integrity of cells, resulting in genetic instability. The article by Jia *et al*.^[17]^ touches upon this important topic of understanding epigenetic changes in the centrosome as relevant to cancer drug resistance.

The focus on immune cells-cancer cells interactions and the reprograming of cancer cells enabling evasion of host immune system over the past several years has brought cancer immunology to the forefront of cancer research^[18]^. In particular, immune checkpoints block the ability of the immune system to act on cancer cells. Thus, immune checkpoint inhibitors have been evaluated to boost up the immune system and potentiate T-cells to recognize and attack tumor cells. As seen in almost all anti-cancer therapies, resistance against immune checkpoint inhibitors has also been reported. Peixoto *et al*.^[19]^ described the epigenetic modifications that underline the resistance against immunotherapy.

Although methylation and acetylation are classical epigenetic events, regulation through miRNAs is also considered an example of epigenetic modification. Further, because of the importance of miRNAs in the regulation of several physiological processes, their expression is itself tightly controlled, often through epigenetic regulation^[20-22]^, thus making miRNAs an important part of cellular epigenetic machinery. The article by Biersack^[23]^ touches upon the role of miRNAs in resistance to alkylating anticancer agents, with a focus on both natural and synthetic alkylating agents and their interactions with miRNAs.

In summary, this Special Issue on the epigenetic basis of cancer drug resistance touches upon some very diverse and hot topics that detail the current progress in understanding the importance of epigenetic events in determining the resistance against various therapies and in a number of different cancers. Without such understanding, the design of novel therapies to counter drug resistance will be impossible.

## Declarations

### Copyright

© The Author(s) 2020.
